# Endogenous tassel-specific small RNAs-mediated RNA interference enables a novel glyphosate-inducible male sterility system for commercial production of hybrid seed in *Zea mays* L.

**DOI:** 10.1371/journal.pone.0202921

**Published:** 2018-08-23

**Authors:** Heping Yang, Youlin Qi, Mike E. Goley, Jintai Huang, Sergey Ivashuta, Yuanji Zhang, Oscar C. Sparks, Jiyan Ma, Brook M. van Scoyoc, Amy L. Caruano-Yzermans, Jennifer King-Sitzes, Xin Li, Aihong Pan, Martin A. Stoecker, B. Elizabeth Wiggins, Marguerite J. Varagona

**Affiliations:** Monsanto Company, St. Louis, Missouri, United States of America; Henan Agricultural University, CHINA

## Abstract

Hybrid crops produce higher yields than their inbred parents due to heterosis. For high purity of hybrid seeds, it is critical to eliminate self-pollination. Manual or mechanical removal of male parts (such as detasseling in maize) is labor-intensive, fuel and time-consuming, and can cause physical damage to female plants, resulting in significant seed yield reductions. Many male-sterility systems either require a maintainer for male-sterile line propagation or are often affected by environmental factors. Roundup^®^ Hybridization System (RHS) utilizes glyphosate to induce male sterility, which effectively eliminates the need for maintainer lines and removal of male parts for commercial hybrid seed production. The first-generation RHS (RHS1) is based on low expression of a glyphosate-insensitive 5-enolpyruvylshikimate-3-phosphate synthase (CP4 EPSPS) in pollen. This report presents the second-generation RHS (RHS2) technology built on RNA interference (RNAi) combined with CP4 EPSPS. It utilizes maize endogenous male tissue-specific small interfering RNAs (mts-siRNAs) to trigger cleavage of the CP4 EPSPS mRNA specifically in tassels, resulting in glyphosate-sensitive male cells due to lack of the CP4 EPSPS protein. Male sterility is then induced by glyphosate application at the stages critical for pollen development, and the male-sterile plants are used as the female parent to produce hybrid seed. The endogenous mts-siRNAs are conserved across maize germplasms, and the inducible male sterility was replicated in representative germplasms through introgression of a CP4 EPSPS transgene containing the mts-siRNA target sequence. This technology combines the relative simplicity and convenience of a systemic herbicide spray methodology with targeted protein expression to create an inducible male sterility system for industrial production of row crop hybrid seeds in an environmentally-independent manner.

## Introduction

Hybrid crops derived from crosses between diverse varieties of a species or different species exhibit greater growth rate, biomass, fertility and yield than their parents due to heterosis [1]. Hybrids account for more than half of major crops cultivated worldwide [2]. In the USA, most of the planted maize acreage is hybrids [3]. For high purity of hybrid seeds, it is critical to eliminate self-pollination. Cytoplasmic male sterility [2]), genic male-sterility mutants [4, 5], transgenic lines in which proteins toxic to male tissues are expressed [6–9], or transgenic lines in which genes critical to male tissue development are suppressed [10–13] can be used to prepare female parents for hybrid seed production. However, these male sterility systems require one or more maintainers for fertility restoration so that the male sterile line can be propagated [2, 14, 15].

Co-expression of two nonoverlapping fragments of a phytotoxic barnase gene inserted separately on allelic positions of the same chromosome can eliminate the need of restoration lines, but this split-gene system still requires a fertile maintainer and only 50% of the resulting progeny will be male-sterile [16]. Environment-sensitive genic male-sterility mutants enable a two-line system because the same mutants can serve as a maintainer under permissive conditions, but this system is environment-dependent [2, 17]. In chemically induced male sterility systems, gametocides can cause damage to non-male tissues because of poor selectivity [18, 19], and male parents must be protected to avoid injury to the stamens when female parents are sprayed with a chemical hybridization agent.

Manual or mechanical removal of male parts (such as detasseling) is a common practice in hybrid maize seed production but is labor-intensive, fuel and time-consuming, and can cause physical damage to female plants, resulting in significant seed yield reductions [20]. Although effective, mechanical detasseling can be imperfect, and hybrid seed purity may be at risk without multiple rounds of manual detasseling. Therefore, an inducible male sterility system that combines the relative simplicity and convenience of chemical methods with the effectiveness and specificity of the transgenic approach are of great value for industrial production of row crop hybrid seeds in an environmentally independent manner.

Glyphosate, best known for its broad spectrum herbicidal activity against weeds, exhibits low mammalian toxicity and a favorable environmental profile [21]. Roundup^®^ and other glyphosate-based herbicides target 5-enolpyruvylshikimate-3-phosphate synthase (EPSPS), a shikimate pathway enzyme required for plant survival. Roundup Ready^®^ (RR) crops contain a transgene encoding a glyphosate-insensitive EPSPS derived from *Agrobacterium* sp. strain CP4 (CP4 EPSPS) that confers tolerance to glyphosate [22].

Unlike other herbicides, glyphosate is a highly systemic herbicide that is readily translocated from source to sink tissues, such as apical meristems in plants [23, 24]. Due to the slow rate of glyphosate metabolism in maize, applications prior to and during the development of the male reproductive tissue could result in the presence of glyphosate in these tissues and thus prevent pollen development, pollen shed, or anther extrusion. Based on this unique feature of glyphosate, the robust, environmentally-independent, inducible Roundup^®^ Hybridization System (RHS) was established for industrial applications in row crops. The first generation RHS (RHS1) relies on a combination of promoter and intron to drive expression of CP4 EPSPS preferentially in vegetative and female reproductive tissues with very low expression in pollen, resulting in glyphosate-sensitive male tissues [21]. Male sterility is then induced by application of glyphosate herbicide at late growth stages [25].

RNA interference (RNAi) is a biological process in which small noncoding RNAs trigger post-transcriptional gene silencing [26]. RNAi is present in many eukaryotes including plants and is a powerful tool to regulate gene expression [27]. Both microRNAs (miRNAs) and small interfering RNAs (siRNAs) can trigger RNAi [28]. External siRNAs, synthesized *in vitro* or generated *in vivo* from dsRNA delivered by a transgene, have been widely used to suppress endogenous genes of interest [29]. Transgenes bearing miRNA-responsive sensor sequences complementary to miRNAs in the 3’ untranslated region (3’UTR) have been used to investigate endogenous miRNAs expression [30].

Since miRNA-mRNA recognition does not require perfect pairing, one miRNA potentially can recognize an array of targets and many miRNAs in fact have multiple targets [31, 32]. In contrast, siRNA-mRNA recognition is a highly-specific paring with only one siRNA target recognized per siRNA [29]. An miRNA typically has just one recognition site on its target, while siRNA has multiple recognition sites spread across the target for a multitude of siRNAs [28, 32]. In this study, endogenous siRNA-mediated RNAi was utilized to develop a second-generation RHS (RHS2) technology, in which an siRNA target sequence was placed in the 3’ untranslated region (UTR) of the CP4 EPSPS transgene, resulting in CP4 EPSPS protein expression to confer glyphosate tolerance in all maize tissues except tassels. The hypothesis is that endogenous siRNAs can be used to regulate expression of a transgene in plants. Specifically, endogenous male tissue-specific siRNAs (mts-siRNAs) recognize transgenic mts-siRNA targets and trigger cleavage of the CP4 EPSPS mRNA only in maize tassels. This can be used in conjunction with timed application of glyphosate for inducible male sterility system useful for hybrid seed production on an industrial scale.

## Materials and methods

### Transformation and event selection

A binary construct was designed containing four transgenic cassettes (**S2 Fig**) for the tolerance to inhibitors of acetyl CoA carboxylase (ACCase) in the aryloxyphenoxy propionate (FOPs) group such as quizalofop; synthetic auxins such as dicamba and 2,4-D; inhibitors of glutamine synthetase such as phosphinothricin or glufosinate; and the EPSPS inhibitor glyphosate. The RHS2 cassette contained the CP4 EPSPS transgene driven by the CaMV 35S promoter. To enable inducible male sterility by glyphosate application, an mts-siRNA target sequence was placed between the CP4 EPSPS stop codon and 3’UTR. Transgenic plants were generated using the binary construct through *Agrobacterium tumefaciens* (ABI strain)-mediated transformation of immature embryos derived from an inbred line of maize (*Zea mays* L.) and then subjected to a series of screenings and molecular characterizations, basically as previously described [25], to identify backbone-free events carrying a single copy of the intact T-DNA. The maize germplasm that was used for the transformation is referred to as wild type (WT) and used as a control hereafter.

Conventional and homozygous transgenic inbred plants were grown for agronomic trials in fields at various locations or for molecular experiments in a greenhouse with 14 hours of natural sunlight supplemented with an irradiance of 50–120 W m^-2^ (provided by 1000 W lamps) at 25°C and 8 hours of dark at 19°C and 70% relative humidity. Plants were watered as necessary and fertilized with a 20-20-20 mixture of nitrogen, potassium, and phosphorus, respectively.

### Genomic DNA extraction

Leaf tissues were ground in liquid nitrogen to a fine powder. Genomic DNA was extracted from the powder using the cetyltrimethylammonium bromide (CTAB) method [33]. DNA was precipitated with ethanol and rinsed in 70% ethanol. The DNA pellet was dissolved in TE buffer (10 mM TRIS, 1 mM EDTA, pH 8.0), and the quality and quantity of the DNA samples were assessed using a NanoDrop 1000 instrument (Thermo Fisher Scientific, Waltham, MA) and stored in a -20°C freezer until use.

### Characterization of endogenous genes

A pair of forward and reverse primers, G1158118 GF1 and G1158118 GR1 (**S1 Table**), was designed to amplify a genomic fragment that shares sequence similarity with a pool of mts-siRNAs. Polymerase chain reaction (PCR) was conducted in a mixture that contained 180 ng of genomic DNA from 31 maize inbred lines as templates, 2.5 units of Ex Taq DNA polymerase (Takara Bio Inc., Kusatsu, Shiga, Japan), and 20pmoles each of the forward and reverse primers on a Thermal Controller (MJ Research Inc., Waltham, MA) programmed as follows: after initial denaturation of 30 sec at 95°C, 35 cycles of amplification (each consisting of a denaturation step of 10 sec at 95°C, an annealing step of 30 sec at 55°C, and an extension step of 35 sec at 72°C). PCR products were purified using a QIAquick Nucleotide Removal Kit (Qiagen, Hilden, Germany) and then sequenced using the forward and reverse primers, respectively, with a Taq Dye Deoxy Terminator Cycle Sequencing Kit on a 373A DNA sequencer (Thermo Fisher Scientific).

### Total RNA extraction

Plant samples were collected at indicated stages, frozen immediately in liquid nitrogen, and individually ground in liquid nitrogen to a fine powder. Total RNA including small RNAs was extracted by using RNA Purification Kit (Norgen Biotek Corp., Thorold, ON, Canada) for microarray or TRIzol^®^ Reagent (Thermo Fisher Scientific) for other assays. The quality and quantity of the RNA samples were assessed using a NanoDrop 1000 instrument (Thermo Fisher Scientific), and the RNA samples were stored in a -80°C freezer until use.

### Small RNA sequencing

Small RNAs were purified with the mirPremier microRNA Isolation Kit (Sigma, St. Louis, MO) from RNA samples extracted from ear, leaf, root, silk, and tassel tissues collected at various growth stages. Libraries were prepared from the small RNA samples with a TruSeq Small RNA Library Preparation Kit (Illumina, San Diego, CA), and sequencing of the libraries was performed on a MiSeq (Illumina).

### Microarray

The microarray chips contained triplicate probes of the complementary sequence for each of the selected 1,216 mts-siRNAs from tassel tissues. Total RNAs isolated from 26 WT tissues collected from 31 germplasms were used to synthesize complementary DNA (cDNA). Cy5-labelled complementarity RNAs were then prepared from the cDNA samples and hybridized with custom microarray chips (LC Sciences, Houston, TX). Hybridization images were collected using a GenePix^®^ 4000B laser scanner (Molecular Devices, Sunnyvale, CA) and digitized using Array-Pro^®^ Analyzer image analysis software (Media Cybernetics, Rockville, MD). Relative signal values were derived by background subtraction and normalization. Differentially expressed signals were determined by t-test with p < 0.05.

### Real-time PCR

Total RNA was used for expression analyses of endogenous genes by a TaqMan or a SYBR Green assay (**S1 Table**). One-step real-time PCR was performed in singleplex with three replicates for each RNA sample. Maize elongation factor 1a (EF1a) was used as a normalizer. Relative expression levels were determined as previously described [34].

### Rapid amplification of 5’ cDNA ends (5’RACE)

Total RNA from tassel, leaf, and ear samples was treated with the TURBO DNase I (Thermo Fisher Scientific) to remove any potential trace of genomic DNA. After phenol/chloroform extraction and ethanol precipitation, 5’RACE was conducted using a transgene-specific primer upstream of a predicted poly(A) site and a nested transgene-specific primer, T-Os.GRP3 GSP11 and T-Os.GRP3 GSP21 (**S1 Table**), with the 5' RACE System (Thermo Fisher Scientific) following the procedure provided by the manufacturer. The extension time for the uncleaved transcript (>2.1 kilobase pairs (kb)) was 2 min 10 sec and for the cleavage products (<400 base pairs (bp)) was 25 sec. The products were purified using the QIAquick Nucleotide Removal Kit (Qiagen), subcloned into the pCR^TM^2.1-TOPO vectors using a TOPO^®^ TA Cloning Kit (Thermo Fisher Scientific), and then sequenced using the pCR Seq Primer 101 or 201 (**S1 Table**), respectively, with a Taq Dye Deoxy Terminator Cycle Sequencing Kit on a 373A DNA sequencer (Thermo Fisher Scientific) in accordance with the manufacturer’s protocols.

### Bioinformatics analysis

Differential bioinformatic analysis was conducted to identify tassel-enriched small RNA sequences by comparing the sequences in the tassel small RNA libraries with those in the small RNA libraries prepared from other maize tissues. Sequence alignment tools such as Basic Local Alignment Search Tool or SHort Read Mapping Package [35] were employed to identify putative endogenous targets that have clustered, overlapping alignments of multiple mts-siRNA sequences with perfect or near perfect matches by comparing the mts-siRNA sequences against unigene collections of maize cDNA sequences. To identify cleavage sites, sequence alignments of the CP4 EPSPS transgene and 5’RACE products were generated using Multalin [36] after the adaptor, poly(C) tail, and vector sequences were removed manually from the sequences of 5’RACE products.

### Low molecular weight RNA blotting

Total RNA from each sample, a 1kb RNA Ladder (Thermo Fisher Scientific) combined with the 25-nt CP4 EPSPS RNA oligos, or the size markers were mixed with the RNA loading Buffer (Ambion, Grand Island, NY), denatured at 95°C for 5 min followed by 4°C for 5 min, loaded to wells of a Criterion Precast 15% polyacrylamide TBE-Urea gel, separated by electrophoresis in a Criterion Cell, and transferred onto positively charged nylon membrane via electroblotting with a Criterion Blotter (Bio-Rad, Hercules, CA) following the manufacture's instruction.

Using T-DNA templates and three sets of the gene-specific primers (**S1 Table)**, three fragments covering the entire CP4 EPSPS transcript except the mts-siRNA target sequence (to avoid cross hybridization with the endogenous mts-siRNAs) (**S2 Fig**) were obtained by PCR amplifications using Ex Taq DNA polymerase (Takara Bio Inc.) and purified with the QIAquick Gel Extraction Kit (Qiagen). DIG-labeled probes CP4 EPSPS 5’, CP4 EPSPS 3’, and OsGRP3 3’UTR were prepared from the PCR products with a PCR DIG Probe Synthesis Kit (Roche, Basel, Switzerland). A 25-nt RNA oligo with a sequence (GGGUGCUGACAUCGAGGUUAUCAAU) identical to a portion of the CP4 EPSPS mRNA for use as a positive control and two DIG-labeled 20- and 25-nt RNA oligos for use as size markers were synthesized. Hybridizations were then conducted at 38°C with rotation in DIG Easy Hyb (Sigma). Post-hybridization washes were done as recommended by the manufacture, and DIG detection was performed using reagents and Lumi-Film X-ray films from Roche. In addition, the RNA blot was rinsed in RNase-free water briefly, incubated in the Stripping Buffer (0.2% SDS and 0.1× SSC) for 45 min at 70°C twice to remove the DIG-labeled probes, equilibrated in 2× SSC, and then reprobed with a synthetic DIG-labeled 21-nt DNA oligo (DIG-CAGAGCTCCCTTCAATCCAAA) designed for detection of zm-miR159b as a control.

### Pollen viability assessment

Plants were sprayed with glyphosate in a greenhouse at the V3 stage or sequentially at V3, V8, and V10. Tassels were harvested from 10 sprayed and 10 unsprayed plants when fully tolerant plants had around 50% of their anthers extruded. Ten anthers at full pollen shed from each tassel were collected, and pollen viability was tested by using Alexander’s stain [37]. The remaining anthers from each tassel were placed in a 150-ml beaker containing 70 ml of 8.5% mannitol solution (pH 5.5) and blended with an Osterizer Classic Chrome Beehive Blender (Oster, Hilliard, Ohio) for 2 min. The mixture was sieved to a 250-ml beaker using a 100 μm sieve twice. The pollen solution was transferred from the beaker to a 50-ml screw top tube, 8.5% mannitol solution (pH 5.5) was added to 50 ml, and the mixture was centrifuged at 1000 ×g for 5 min. The supernatant was carefully removed, and the pellet was re-suspended in 30 ml 8.5% mannitol solution (pH 5.5) and mixed well by swirling. Immediately, 15 μl of the pollen solution was loaded to the V-shaped groove on a hemocytometer (Hausser Scientific, Horsham, PA), and then pollen grains (mature and non-mature) on all nine squares (full grid) of the hemocytometer were counted. The highest and lowest pollen grain scores were discarded before calculation for the total number of pollen.

## Results

### Identification of endogenous mts-siRNAs

In the current study, endogenous male tissue-specific small interfering RNAs (mts-siRNAs) were defined as being 18 to 26-nucleotides long, with some partially overlapping or complementary to others. Maize mts-siRNAs were identified and used to post-transcriptionally suppress expression of a CP4 EPSPS transgene in a tissue-specific manner.

**Fig 1:**
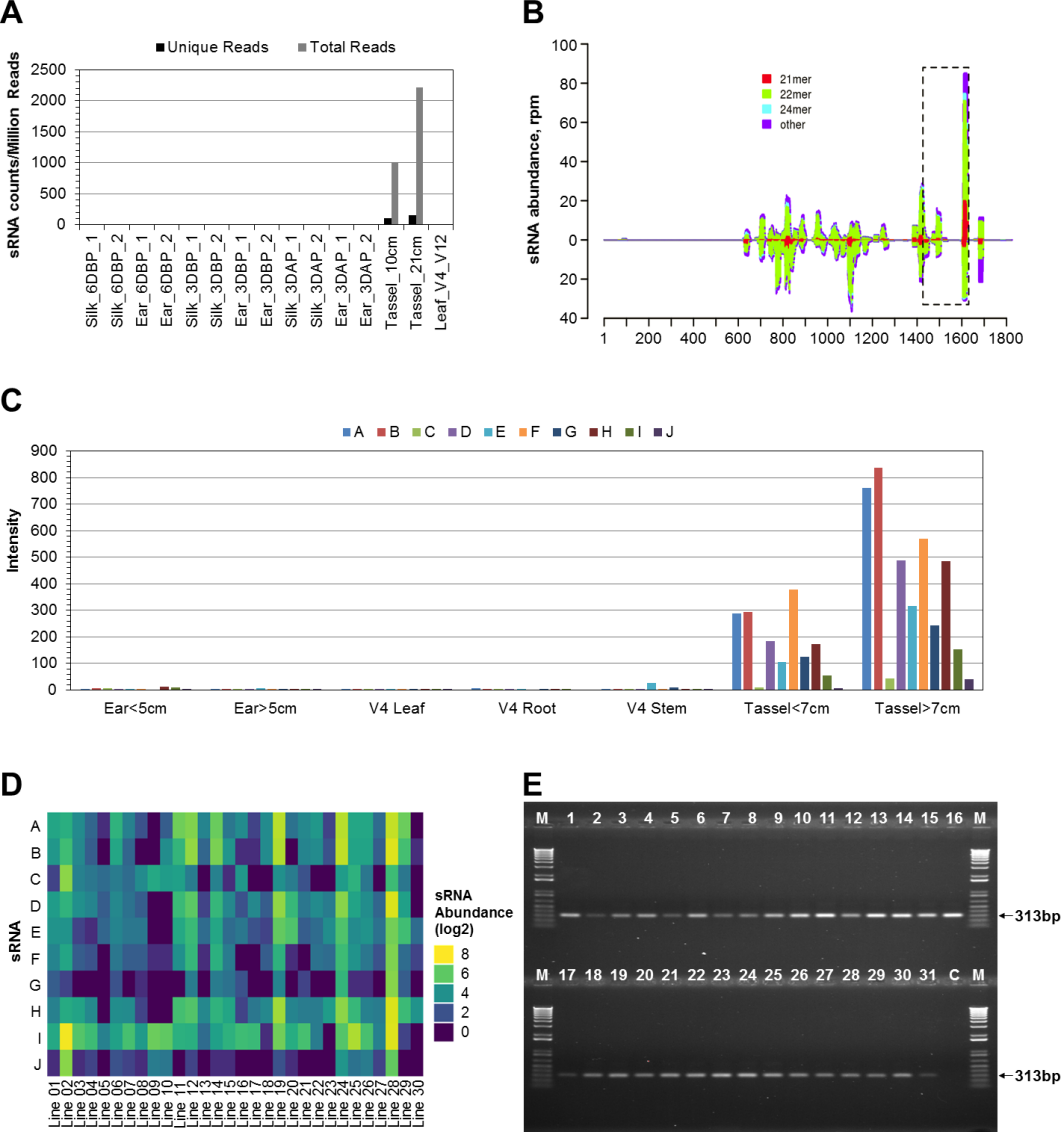
Identification of mts-siRNAs and selection of transgenic target. (**A**) Abundance of putative EU974548 targeting mts-siRNAs in small RNA libraries derived from 15 tissues as indicated. Unique reads do not include redundant reads. Total reads count both unique and redundant reads. DBP: days before pollination; DAP: days after pollination. (**B**) Distribution of mts-siRNAs on the EU974548 cDNA sequence (1826 bp long). mts-siRNAs sharing the same as or complementary to the cDNA strand are shown above and below the zero line, respectively. The vertical axis indicates normalized relative expression levels of mts-siRNAs. The horizontal axis shows the nucleotide position from the beginning of the cDNA. mts-siRNA lengths are indicated by different colors. The boxed area, rich in mts-siRNA recognition sites, was selected as a transgenic target and placed after the coding region of the CP4 EPSPS (**S2 Fig**). (**C**) Tassel specificity of representative mts-siRNAs targeting the boxed area. Abundances of mts-siRNAs A to J (**S2 Table.**) in maize tissues of the germplasm into which the transgene was transformed were measured by microarray. The vertical axis shows signal intensity. (**D**) Presence of the mts-siRNAs A to J in V8 tassels of thirty additional representative maize germplasms commonly cultivated in North America. Graphic representation was based on microarray signal intensities. (**E**) Conservation of one endogenous gene (*Zm00001d036860*) with sequence homology to the mts-siRNAs. Lane M: 1kb Plus DNA size marker (Thermo Fisher Scientific). Lanes 1 through 31: PCR products from genomic DNA obtained from leaves of the germplasm into which the transgene was transformed and thirty additional representative maize germplasms commonly cultivated in North America. Lane C: no templates control.

To identify mts-siRNAs, small RNA libraries prepared from various maize tissues were subjected to deep sequencing. Small RNA sequences obtained from tassels at different developmental stages were compared with those from other organs including leaves, roots and ears. Thousands of small RNAs that are primarily expressed in tassel tissues were identified, with normalized expression levels ranging from 40 to 2660 transcripts per million reads. As an example, **Fig 1A** shows the normalized abundance of one cluster of such small RNAs in one set of the libraries prepared from 15 maize organs.

From the tassel-enriched small RNAs, 1,216 putative mts-siRNAs were chosen for further evaluation using custom microarray technology. Microarray data from custom chips designed for detection of the selected mts-siRNAs revealed that more than 500 mts-siRNAs exhibited tassel-specific expression in a panel of organs from WT plants, as shown in **Fig 1C** for ten such mts-siRNAs (**S2 Table**). Additional microarray experiments indicated that the majority of the mts-siRNAs were present in tassels from 30 other inbred germplasms tested (**Fig 1D**).

### Identification of endogenous genes with sequence homology to mts-siRNAs

An endogenous gene with sequence homology to mts-siRNAs may be responsible for biogenesis of the siRNAs and may be the target for the siRNAs. To identify endogenous genes with sequence homology to mts-siRNAs, over 500 mts-siRNAs were aligned against the cDNA sequences in unigene collections from maize. More than a dozen cDNA sequences were found containing one or more regions rich in potential mts-siRNA targets with perfect or near perfect sequence matches. One of these, EU974548, was previously isolated from a maize hybrid via large-scale sequencing of expressed sequence tags [38]. A graphical representation of the alignment of mts-siRNA and EU974548 sequences is shown in **Fig 1B**. The mts-siRNA sequences aligned to either sense or antisense strand of the EU974548 cDNA sequence.

To determine whether the EU974548 sequence is present in diverse maize germplasms, PCR amplification was conducted using genomic DNA as a template. Products with the expected size of 313bp were obtained from 31 representative maize germplasms cultivated in North America (**Fig 1E**). Sequencing results of these amplicons revealed that they share over 92% sequence identity with the EU974548 cDNA (**S1 Fig**). Moreover, bioinformatic searches using multiple sequence databases confirmed that the EU974548 sequence is present in representative germplasms cultivated in global regions across the world, indicating the EU974548 sequence is broadly conserved in maize.

### Selection of commercial transgenic events

The conservation of the mts-siRNAs (**Fig 1D**) and the EU974548 sequence (**Fig 1E**) across a wide range of maize germplasms makes EU974548 ideal for use in transgene regulation. Surveying the cDNA sequence alignment, several notable regions that display clustered, overlapping alignments of multiple mts-siRNA targets were identified. A 201-bp region was identified as having multiple potential recognition sites corresponding to the most abundant mts-siRNAs (**Fig 1B**) was used to create synthetic mts-siRNA target sequences. A construct was made with the element placed 3’ to the coding region of the CP4 EPSPS gene (**S2 Fig**). Transgenic plants were generated from the binary vector via *Agrobacterium* (ABI strain)-mediated transformations. Subsequently, homozygous plants were evaluated for effectiveness of glyphosate-induced male sterility. After initial testing, the 201-bp mts-siRNA version was found to be efficacious for suppressing transgene expression in male reproductive cells.

Maize immature embryos were transformed by *Agrobacterium* carrying the four-cassette construct including the CP4 EPSPS transgene linked to the 201-bp mts-siRNA target sequence (**S2 Fig**), producing 436 transgenic events of which 92 were backbone-free and carrying only a single copy of the insertion in plants. In the greenhouse, 70 of the 92 events demonstrated tolerance to glyphosate. Of these, 30 events were advanced to field testing where 22 provided vegetative tolerance to glyphosate and inducible male sterility. Additional field trials, trait efficacy trials, and molecular characterizations were then conducted for multiple years in order to select the commercial event MON 87429 that contains the RHS2 trait.

### Tassel-specific cleavage of the CP4 EPSPS mRNA with mts-siRNA recognition sites

**Fig 2:**
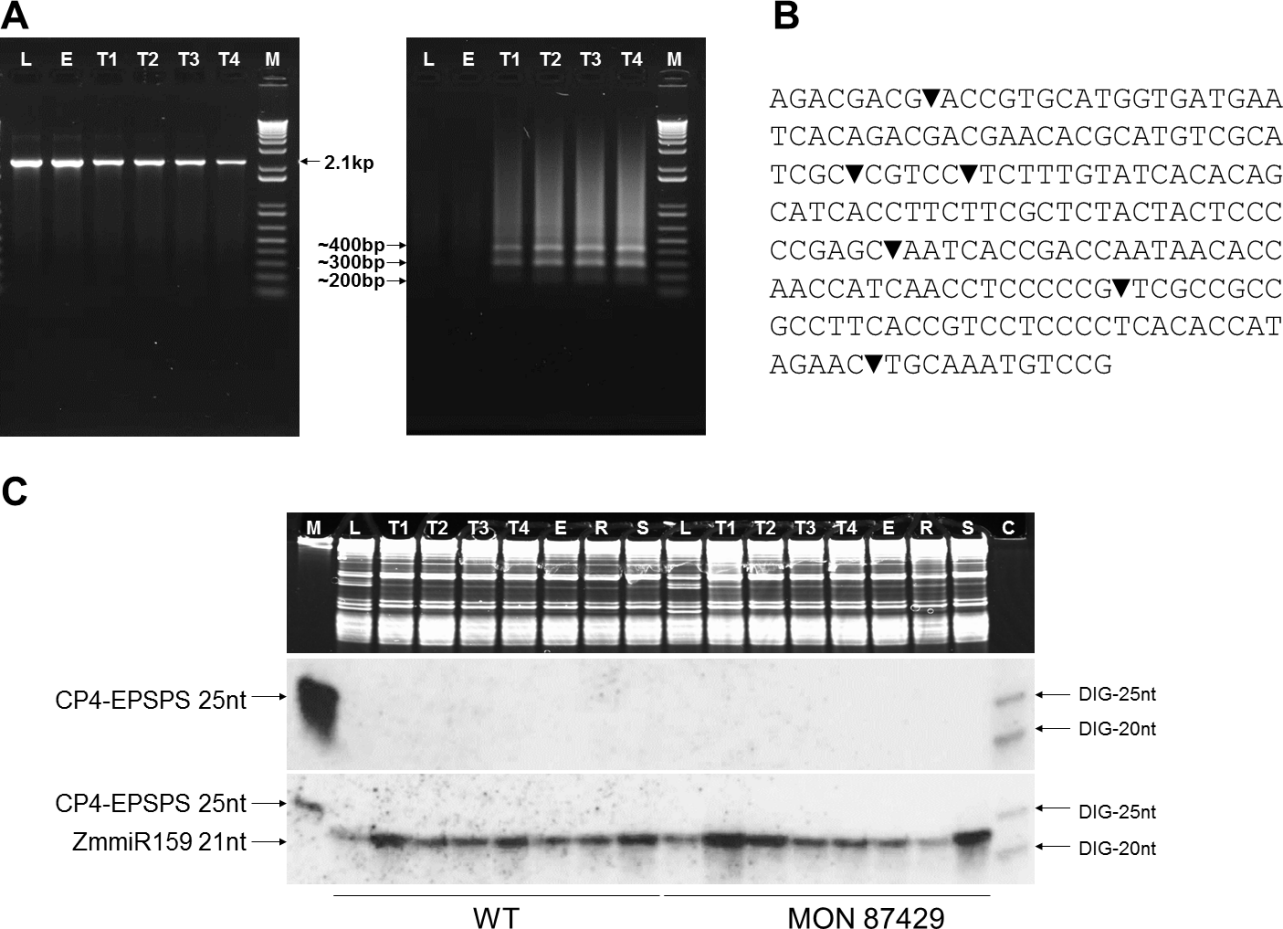
Selective expression of the CP4 EPSPS in plants containing MON 87429. (**A**) Male tissue-specific cleavage of the CP4 EPSPS transcript. Full length CP4 EPSPS (approximately 2.1 kb) was detected in all tissues while cleaved products (<400 bp) were detected only in tassels from plants containing MON 87429. The extension time was 2 min 10 sec for the uncleaved transcript or 25 sec for cleavage products, respectively. M: 1kb Plus DNA size marker (Thermo Fisher Scientific); L: V6 leaf; E: VT ear; T1: V6 tassel; T2: V8 tassel; T3: V10 tassel; T4: V12 tassel. (**B**) Sequence-specific cleavage of the CP4 EPSPS. Cleavage sites (▼) were identified on the mts-siRNA target sequence (201 bp) in the CP4 EPSPS transcript by sequencing the 5’RACE products. (**C**) Absence of CP4 EPSPS small RNAs. Total RNA (10 μg) from various tissues of WT (left) or plants containing MON 87429 (right), respectively, was used for RNA blotting. The upper shows equal loading and quality of the RNA samples by sharp, distinct ribosomal RNA bands in TBE-urea denaturing polyacrylamide gel (ethidium bromide stained). M: a mix of the 1kb RNA ladder (1 μg) and a synthetic 25-nt CP4 EPSPS RNA oligo (1 ng) as a positive control; T1: V6 tassel; T2: V8 tassel; T3: V10 tassel; T4: V12 tassel; E: VT ear; L: V6 leaf; R: VE root; S: VE shoot; C: synthetic DIG-labeled 20- and 25-nt RNAs as small size markers. The middle represents the low molecular weight RNA blot that was hybridized with three DIG-labeled probes covering the whole CP4 EPSPS transcript except the 201-bp target region. The lower illustrates the small RNA blot that was reprobed for miR159 after stripping off the CP4 EPSPS probes to demonstrate small RNA quality of in the samples. VE: emergence stage; Vn: leaf stages by number of leaves on the maize plant; VT: tasseling stage.

Under the control of the cauliflower mosaic virus (CaMV) 35S promoter, the CP4 EPSPS transgene is transcribed throughout the plant. Analysis using 5’ rapid amplification of cDNA ends (5’RACE) of plants containing MON 87429 showed a 2.1-kb product, representing the full-length CP4 EPSPS transcript, in all tissue samples (**Fig 2A**, left) including tassels, and sequencing results confirmed the product was derived from the intact CP4 EPSPS mRNA. Small products, mostly about 300 bp and 400 bp, representing cleavage products of the CP4 EPSPS transcript were obtained only from the tassel and not found in leaf or ear tissue of plants containing MON 87429 (**Fig 2A**, right). Sequencing of these products revealed endoribonuclease cleavage sites exclusively in the mts-siRNA target sequence (**Fig 2B**), indicating that cleavage occurs only at the mts-siRNA recognition sites and in a sequence-specific manner.

### Absence of secondary CP4 EPSPS siRNAs

Secondary siRNAs can move from the site of origin and thus silence homologous genes locally or distantly [29]. To determine whether there are secondary siRNAs derived from the CP4 EPSPS transcript in plants containing MON 87429, low molecular weight RNA blotting [39] was conducted. Ribosomal RNA bands were sharp and even across samples, indicating high quality and equal loading of the RNA samples (**Fig 2C**, upper). The RNA molecules were then transferred onto a membrane and hybridized with three probes that cover the entire CP4 EPSPS transcript except for the mts-siRNA target sequence (**S2 Fig**). This region was intentionally left out to avoid its cross hybridization with the endogenous mts-siRNAs.

An *in vitro*, chemically-synthesized 25-nt RNA oligo with a sequence identical to a portion of the CP4 EPSPS mRNA was detected as a positive control (**Fig 2C**, middle left), indicating that the probes worked and hybridization and visualization were successful. No signal was observed in any samples tested (including leaves, roots, tassels at different developmental stages, and ears) for plants containing MON 87429 or the WT plants (**Fig 2C**, middle), demonstrating that no secondary siRNAs were generated from the CP4 EPSPS transcripts in plants containing MON 87429. After stripping off the CP4 EPSPS probes, the blot was hybridized to a synthetic DIG-labeled DNA oligo complementary to zm-miRNA159, which was previously demonstrated to be expressed in all maize tissues [40] and used as a control here. Zm-miRNA159 was detected in all samples (**Fig 2C**, bottom), indicating separation and electroblotting of small RNAs were successful.

### Inducible male sterility in plants containing MON 87429

**Fig 3:**
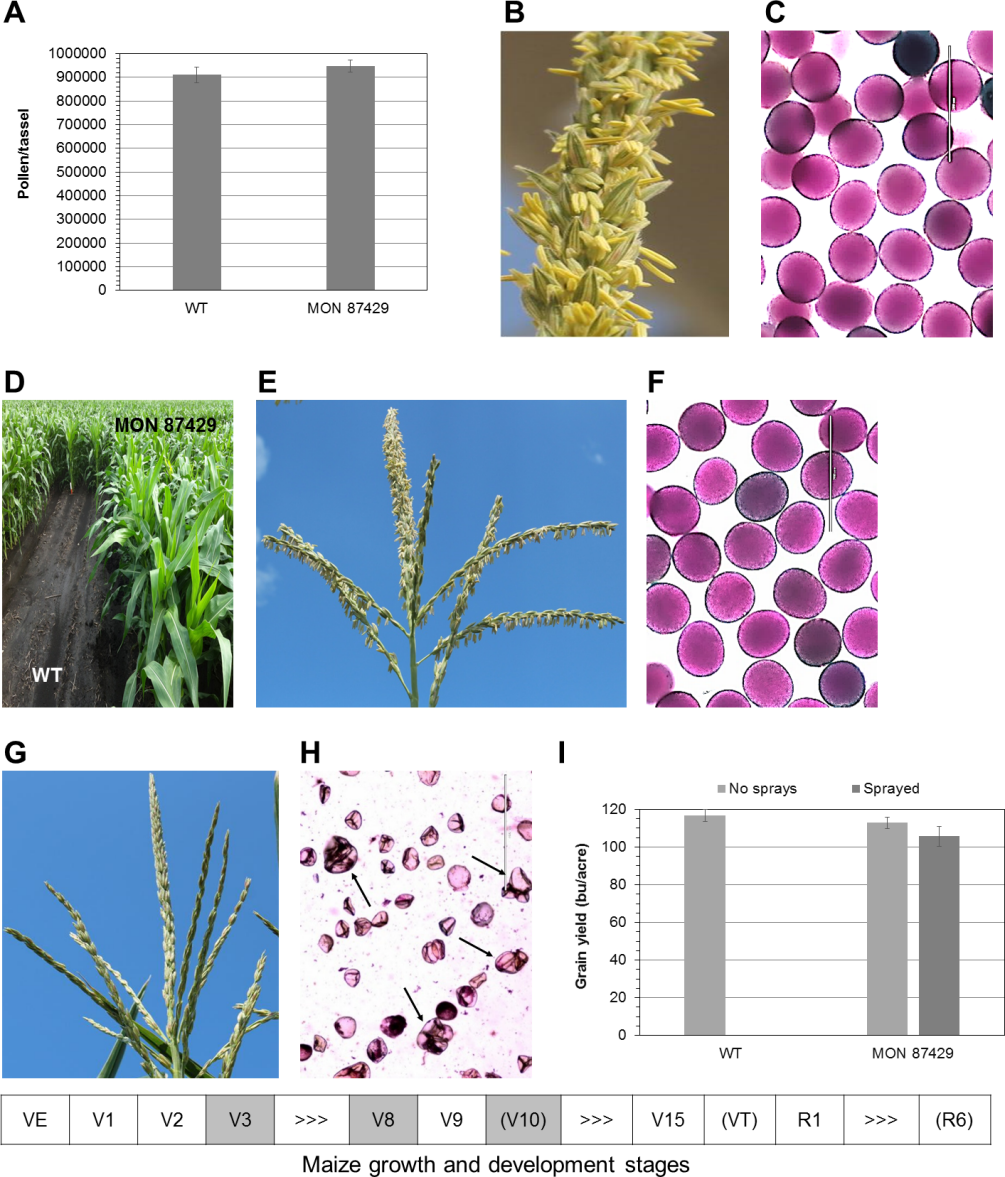
Inducible male sterility in plants containing MON 87429. (**A**) Pollen grain counts. No significant differences in pollen yields were observed between WT plants and plants containing MON 87429 when they were not treated with glyphosate. Whole tassels from 10 plants each were individually harvested in paper bags when pollen shed was observed throughout tassel branches. Pollen, mature or immature, were extracted and counted as described in the methods. (**B**) and (**E**) Tassel fertility of plants containing MON 87429. Anther extrusion was normal on the tassel from a plant that was not treated with glyphosate (**B**), or sprayed with 1.5 lbs ae/acre of glyphosate at stage V3 only for weed control (**E**), respectively. (**C**) and (**F**) Pollen viability of plants containing MON 87429. Pollen were viable when glyphosate was not sprayed (**C**), or applied at stage V3 (**F**), respectively. (**D**) Vegetative tolerance of plants containing MON 87429. No crop injury to the plants was observed after 1.5 lbs ae/acre of glyphosate was applied at stage V3 while WT plants were wiped out along with weeds. (**G**) and (**H**) Inducible male sterility of plants containing MON 87429. Tassels were sterile (**G**) and pollen were not viable (**H**) after 1.5 ae/acre glyphosate was sprayed at stage V3, followed by 0.75 lbs ae/acre each at V8 and V10. (**I**) Grain yields. The least significant difference (LSD) at 0.05 was 6.25 bu/acre between WT counterparts and plants containing MON 87429 that were not treated with glyphosate and 10.50 bu/acre between plants containing MON 87429 or NK603 that were treated with glyphosate at V3, V8 and followed by V10. The symbolic diagram shows maize growth and development stages. Grey shaded: glyphosate applied; in parentheses: data collected [at V10 for (D), VT for (A) to (C) and (E) to (H), and R6 for (I)]; >>>: V4 to V7, V11 to V14, or R2 to R5, respectively. Scale bars = 200 μm in (**C)** and (**F**) or 400 μm in (**H**). Arrows in (**H**) point to tetrads. Vn: leaf stages by number of leaves on the maize plant.

Under normal growth conditions and without glyphosate applications, there was no significant difference in total pollen grain counts per tassel between plants containing MON 87429 and WT plants, the conventional counterpart (**Fig 3A**). Plants containing MON 87429 demonstrated complete fertility with full anther extrusion (**Fig 3B**), viable pollen (**Fig 3C**), and grain yield equivalent to WT plants (**Fig 3I**).

No damage was observed on plants containing MON 87429 when glyphosate was sprayed at vegetative growth stage V3, demonstrating vegetative tolerance (**Fig 3D**). Anther extrusion was normal (**Fig 3E**) and pollen were fully fertile (**Fig 3F**). This enables inbred seed propagation without the need of a maintainer line.

Glyphosate application at stages V3 followed by V8 followed by V10 resulted in no anther extrusion or pollen shedding observed on tassels of plants containing MON 87429 (**Fig 3G**), and more importantly, no viable pollen generated in anthers (**Fig 3H** and **S3 Table**). Pollen development appeared to be arrested at the tetrad stage in these treated plants (**Fig 3H**); however, female reproductive cells were fully fertile and plants containing MON 87429 set seeds normally when cross pollinated by pollen from plants containing NK603 (**Fig 3I**). Plants containing commercial event NK603, which have robust vegetative and reproductive tolerance to glyphosate [22], produced viable pollen after the same glyphosate application (**S3 Table**).

### Expressions of endogenous genes with sequence homology to the mts-siRNA target sequence

Seven putative endogenous genes were identified in the maize genome to have a fragment that shares over 90% identity with the mts-siRNA target sequence via bioinformatics analyses. All these genes are located in one region on chromosome 6. These genes appear to encode putative membrane proteins with amino acid sequence homology to a serine incorporator that facilitates the synthesis of two serine-derived lipids, phosphatidylserine and sphingolipids.

No off-type phenotypes were observed in plants containing MON 87429. To further validate absence of unintended side effects at the molecular level, expression levels of the putative endogenous genes were measured by using two quantitative reverse transcription PCR assays: a TaqMan assay designed for detecting transcripts from two members and a SYBR Green assay designed for six members of the gene family (expression of one member can be detected by both assays). Overall, these genes were expressed at very low levels in both transgenic and WT tassels (**S3 Fig**). Furthermore, the expression levels of the endogenous genes in plants containing MON 87429 were comparable to those in WT plants of the germplasm into which the transgene was transformed and four additional representative inbred lines. Statistically, there were no significant differences between plants containing MON 87429 and WT plants.

## Discussion

**Fig 4:**
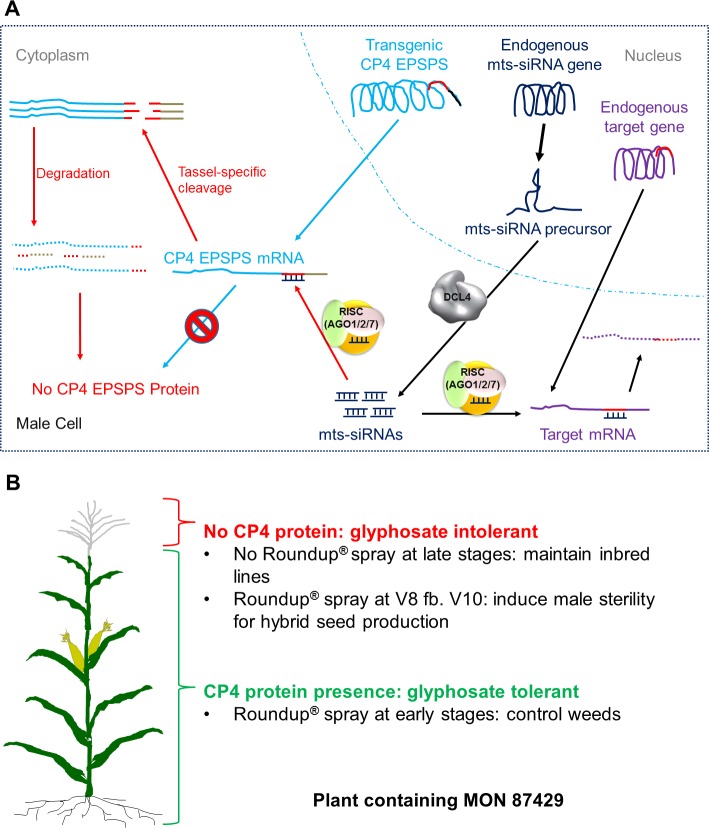
Second generation Roundup^®^ Hybridization System (RHS2). (**A**) Molecular mode of action. Endogenous mts-siRNAs trigger, most likely AGO2-mediated, cleavage of the CP4 EPSPS mRNA in a sequence-specific manner only in male reproductive cells. Lack of protection from a 3’ poly adenine tail or a 5’ cap structure, the cleavage products are then subjected to degradation by 3’-5’ and 5’-3’ exoribonucleases, respectively. As the result, there is little to no CP4 EPSPS protein synthesis in the male reproductive cell. (**B**) Dual-utility. CP4 EPSPS protein is normally accumulated in vegetative and female tissues, providing excellent protection against glyphosate sprays for weed control in the early season, therefore the inbred can be maintained. By contrast, little CP4 EPSPS protein is present in the male reproductive cell. Upon glyphosate sprays at the stages critical for pollen development, male sterility will be induced and the plants can be used as female parent to produce hybrid seeds. AGO: Argonaute; DCL: Dicer-like; RISC: RNA-induced silencing complex.

In this study, siRNA-mediated RNAi was used to selectively suppress transgene expression to create a new inducible male sterility system for hybrid seed production in row crops named RHS2 (**Fig 4**). A key component of the RHS2 technology is a mts-siRNA target sequence is incorporated into the CP4 EPSPS cassette as a unique expression regulatory element within the 3’UTR (**S2 Fig**), and this is then targeted by the endogenous mts-siRNAs (**Fig 4A**). The endogenous mts-siRNAs (**S2 Table**) are tassel-specific (**Fig 1C** and **1D**), so cleavage of the CP4 EPSPS mRNA by the RNAi pathway occurs only in tassels and only in a sequence-specific manner (**Figs 2A** and **4A**). Following the cleavage, 3’ to 5’ and 5’ to 3’ degradations of the cleavage products occur, most likely catalyzed by RNA enzymes such as RNase II and exoribonuclease 1. This results in reduced CP4 EPSPS protein production in male tissues, which in turn leads to glyphosate-sensitive male reproductive cells. Applications of glyphosate at the defined stages thus induce complete male sterility in plants containing MON 87429 (**Fig 3G** and **3H**, **S3 Table** and **Fig 4B**).

Multiple cleavage sites have been identified in the 201-bp mts-siRNA target sequence (**Fig 2B**), demonstrating that the mts-siRNAs (**S2 Table**) trigger target mRNA cleavage specifically at their recognition sites. The 201-bp target has multiple siRNA recognition/cleavage sites, but bioinformatics analyses did not identify any miRNA recognition sites in the sequence. These findings confirm that the RHS2 technology achieves selective gene regulation via the siRNA-mediated RNAi pathway.

The CP4 EPSPS mRNA remains intact in other tissues in plants containing MON 87429 (**Fig 2A**), conferring glyphosate tolerance to the vegetative and female reproductive tissues. Vegetative tolerance of the plants containing MON 87429 allows applications of glyphosate in early season for weed control (**Figs 3D** and **4B**). Without glyphosate sprays, or with glyphosate application only at early vegetative growth stages, both male (**Fig 3C** and **3F**) and female (**Fig 3I**) tissues were fully fertile, which enables inbred seed propagation with no need of a maintainer line. With glyphosate applications timed to induce full male sterility, the plants containing MON 87429 were female fertile and set seeds normally after cross pollination by viable pollen (**Fig 3I**). This demonstrates suitability of plants containing MON 87429 as the female parent for hybrid seed production. Commercial hybrids with complete glyphosate tolerance are generated by crossing a female parent containing MON 87429 with a male parent containing an event for complete glyphosate tolerance, similar to RR commercial hybrids currently in the market [22].

It is crucial that the endogenous mts-siRNAs are present across a broad range of germplasms as possible to achieve optimal germplasm penetrance. The mts-siRNAs accumulated in the tassels of all representative maize germplasms tested (**Fig 1D**). The presence of endogenous genes having a region with highly-conserved sequence homology to the mts-siRNAs in the germplasms (**Fig 1E** and **S1 Fig**) indicates high germplasm penetrance potential for the RHS2 technology. Furthermore, the MON 87429 event was introgressed from the transformation background into 15 other maize germplasms, and all of these displayed male sterility after applications of glyphosate at the designated late growth stages.

Short-distance (cell-to-cell) and long-distance (between organs) movement of small RNAs are associated with spatiotemporal coordination of gene regulation, cell fate decisions, and tissue patterning in multicellular organisms [41, 42]. Secondary siRNAs can amplify post-transcriptional gene silencing locally or distantly in plants (a phenomenon called transitivity) [29]. Small RNA blotting demonstrated that there are no CP4 EPSPS siRNAs present in plants containing MON 87429 (**Fig 2C**), conforming that secondary CP4 EPSPS siRNAs are not produced, and the CP4 EPSPS silencing is constrained to the male cells, so the CP4 EPSPS expression will not be impacted in the other cells. No transitivity is involved so other genes would not be expected to be affected. This is supported by the lack of off types observed in plants containing MON 87429 through many years of field trials.

Built on the RR technology, RHS is a convenient platform utilizing glyphosate to induce male sterility in female parents in a hybrid seed production field [25]. The high sterility standard achieved by RHS2 technology ensures better hybrid seed purity over other male sterility systems. The complete termination of pollen development in a biologically safe and tightly controlled manner and the efficient male fertility restoration in the F1 hybrid plants creates a superior system for hybrid seed production. RHS2 greatly simplifies the process and significantly reduces the cost of commercial hybrid maize seed production at an industrial scale.

Plants containing MON 87429 carry four expression cassettes (**S2 Fig**). The CP4 EPSPS cassette provides vegetative and female tissue tolerance to glyphosate to facilitate hybrid seed production. For weed management the dicamba mono-oxygenase (DMO) and phosphinotricin N-acetyltransferase (PAT) cassettes provide tolerance to dicamba and glufosinate herbicides, respectively. The FOPs and 2,4-D tolerance enzyme variant T (FT_T) cassette provides tolerance to FOPs and some synthetic auxin herbicides. These herbicide tolerant traits provide growers multiple options for weed management utilizing currently available, and widely used, herbicides with varying modes of action.

## Conclusions

The RHS2 technology was created for hybrid seed production to take advantage of the naturally occurring siRNA-mediated RNAi pathway in maize. These mts-siRNAs are specific to maize, however, similar gene expression regulation tools can be developed in other plants by substituting the target sequence with one recognized by mts-siRNAs from the species of interest. This selective gene regulation technology by native siRNAs provides a novel method for fine tuning transgenic expression at the tissue, organ, or cell level in order to achieve desired patterns.

## Supporting information

S1 Fig Sequence conservation of an endogenous gene with sequence homology to the mts-siRNA target sequence. PCR products were obtained from the germplasm into which the transgene was transformed and thirty additional representative maize germplasms commonly cultivated in North America. Sequences were determined by Sanger sequencing. Sequence alignments were generated with a program by Corpet [37] using the default parameters. Nucleotides identical to those of the first sequence are represented by dots.
(TIF)

S2 Fig Transgenic elements in the T-DNA for generating plants containing MON 87429. LB: left border; PAT: phosphinotricin N-acetyltransferase; DMO: dicamba mono-oxygenase; FT_T: FOPs and 2,4-D tolerance enzyme variant T (α-ketoglutarate-dependent dioxygenase); CP4 EPSPS: *Agrobacterium* sp. strain CP4 5-enolpyruvylshikimate-3-phosphate synthase; mts-siRNA: male tissue-specific small interfering RNAs; RB: right border. Not drawn to scale.
(TIF)

S3 Fig Expression of endogenous genes with sequence homology to the mts-siRNAs in V8 tassels. (A) Expression levels of two endogenous genes as determined by a TaqMan assay (S1 Table). (B) Expression levels of six endogenous genes as determined by a SYBR Green assay (S1 Table). Plants containing MON 87429 (test) and WT counterparts (control) and additional 4 inbred lines (reference) were grown in a randomized complete block design with 100 replications for the test and control materials, and 25 replications for each reference material in a greenhouse. Each replication included three technical replicates (subsamples) per material. Real time PCR was conducted in triplicate for each subsample. The statistical analysis using a linear mixed model for a randomized complete block design reveals there are no significant differences out of two comparisons at the 5% level. Black dots: mean; gray dots: upper or lower 95% confidence interval, respectively.
(TIF)

S1 Table Primers and probes used in this study. (DOCX)

S2 Table Representative mts-siRNAs with sequence homology to the mts-siRNA target sequence. (DOCX)

S3 Table Pollen yield per tassel and pollen viability. (DOCX)
