# Supplementary figures and images for: Endogenous tassel-specific small RNAs-mediated RNA interference enables a novel glyphosate-inducible male sterility system for commercial production of hybrid seed in Zea mays L
Source: PLoS One. 2018 Aug 23;13(8):e0202921. doi: 10.1371/journal.pone.0202921 (PMC6107248; doi:10.1371/journal.pone.0202921)

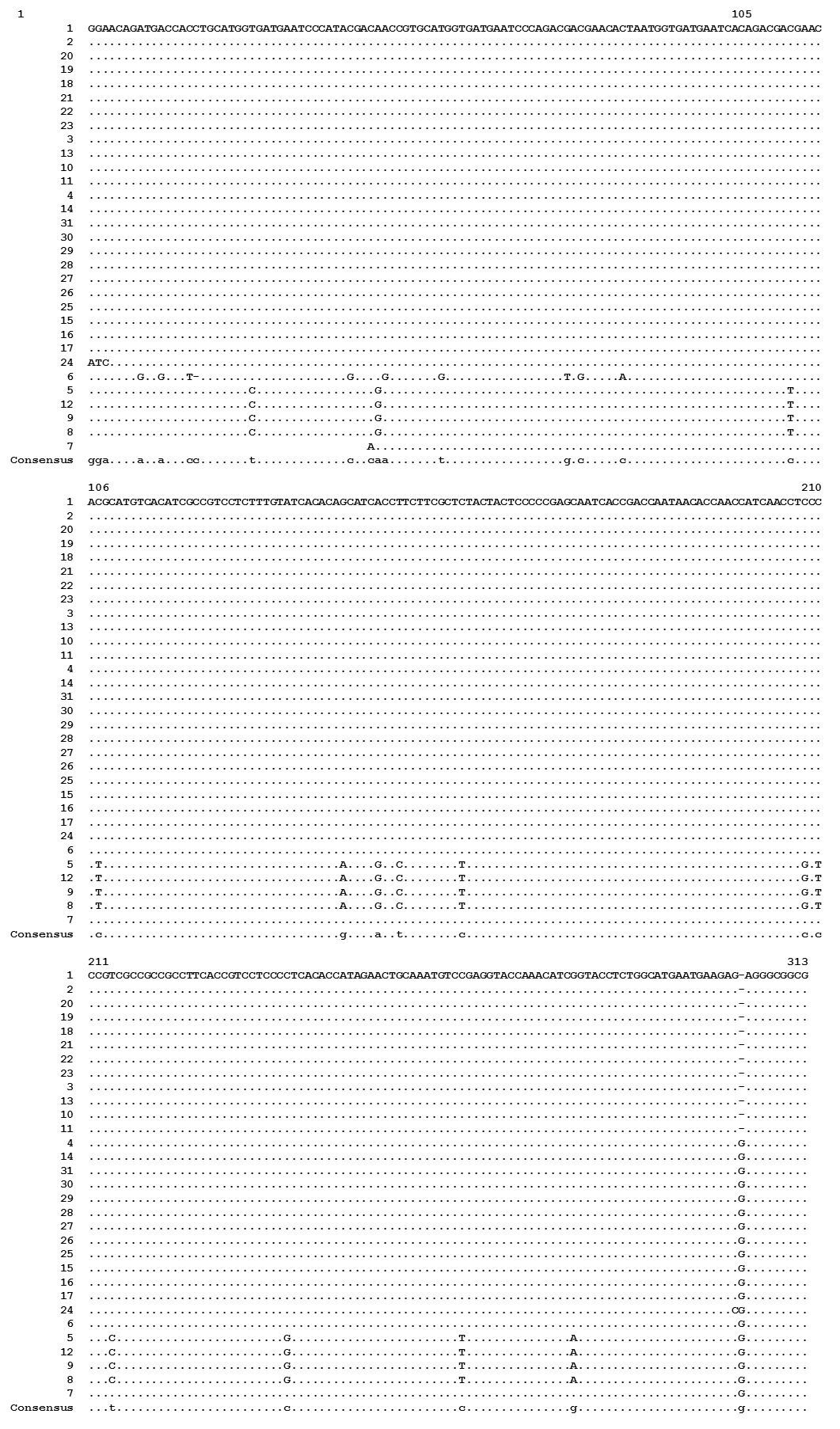

Supplement: S1 Fig — PCR products were obtained from the germplasm into which the transgene was transformed and thirty additional representative maize germplasms commonly cultivated in North America. Sequences were determined by Sanger sequencing. Sequence alignments were generated with a program by Corpet [37] using the default parameters. Nucleotides identical to those of the first sequence are represented by dots. (TIF) [file pone.0202921.s001.TIF]

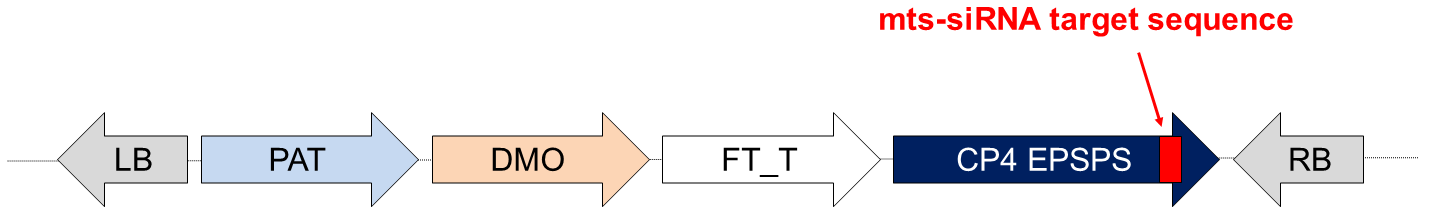

Supplement: S2 Fig — LB: left border; PAT: phosphinotricin N-acetyltransferase; DMO: dicamba mono-oxygenase; FT_T: FOPs and 2,4-D tolerance enzyme variant T (α-ketoglutarate-dependent dioxygenase); CP4 EPSPS: Agrobacterium sp. strain CP4 5-enolpyruvylshikimate-3-phosphate synthase; mts-siRNA: male tissue-specific small interfering RNAs; RB: right border. Not drawn to scale. (TIF) [file pone.0202921.s002.TIF]

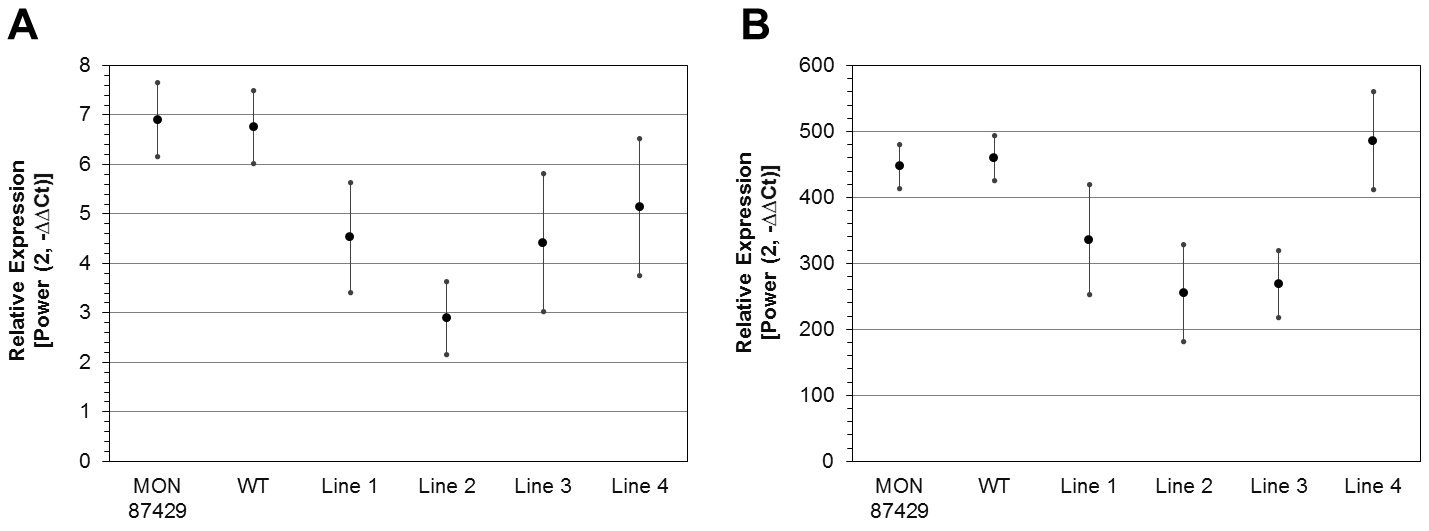

Supplement: S3 Fig — (A) Expression levels of two endogenous genes as determined by a TaqMan assay (S1 Table). (B) Expression levels of six endogenous genes as determined by a SYBR Green assay (S1 Table). Plants containing MON 87429 (test) and WT counterparts (control) and additional 4 inbred lines (reference) were grown in a randomized complete block design with 100 replications for the test and control materials, and 25 replications for each reference material in a greenhouse. Each replication included three technical replicates (subsamples) per material. Real time PCR was conducted in triplicate for each subsample. The statistical analysis using a linear mixed model for a randomized complete block design reveals there are no significant differences out of two comparisons at the 5% level. Black dots: mean; gray dots: upper or lower 95% confidence interval, respectively. (TIF) [file pone.0202921.s003.TIF]
