# Supplementary material for: Endogenous tassel-specific small RNAs-mediated RNA interference enables a novel glyphosate-inducible male sterility system for commercial production of hybrid seed in Zea mays L
Source: PLoS One. 2018 Aug 23;13(8):e0202921. doi: 10.1371/journal.pone.0202921 (PMC6107248; doi:10.1371/journal.pone.0202921)
Supplement: S1 Table — (DOCX) [file pone.0202921.s004.docx]

**S1 Table. Primers and probes used in this study.**

| **Primer ID** | **Sequence** | **Tm** | **Expected product** |
| --- | --- | --- | --- |
| **For detection of an endogenous gene** | | | |
| G1158118 GF1 | GGAACAGATGACCACCTGCAT | 60.07 | 313 bp |
| G1158118 GR1 | CGCCGCCCTCCTCTTC | 42.48 |  |
| **For expression analyses of endogenous genes** | | | |
| Forward Primer 1 | AAACCTACGGGAAAGTTGCG | 61.33 | 105 bp |
| FAM Probe 1 | 6FAM-ACAAGAAATACCCCTGCTCCAACCTG-MGBNFQ | 68.10 |  |
| Reverse Primer 1 | TCTCGCATAATTTGTAGTTCAGCTG | 61.83 |  |
| Forward Primer 2 | TGGATGAATGKAATCTGAACAAGCT | 62.13 | 73 bp |
| Reverse Primer 2 | CGGGGCAMTTGAACAATCCTA | 62.97 |  |
| **For normalization of expression** | | | |
| EF1a Forward Primer | GCTAGCTTTACCTCCCAGGTCATC | 63.55 | 64 bp |
| EF1a VIC Probe | VIC-TCATGAACCACCCTGGC-MGBNFQ | 73.29 |  |
| EF1a Reverse Primer | GGGCATAGCCATTGCCAATC | 64.68 |  |
| **For 5’RACE to identify cleavage sites** | | | |
| T-Os.GRP3 GSP21 | CAACAAGCAGTTCAAACACCAT | 60.07 | >205 bp |
| T-Os.GRP3 GSP11 | TTCTATTTCATTTCATTC | 42.48 | >233 bp |
| pCR Seq Primer 101 | CAGGAAACAGCTATGACCATGAT | 60.35 | NA |
| pCR Seq Primer 201 | CGACGGCCAGTGAATTGTAATAC | 62.70 | NA |
| **For preparation of probes for Northern hybridization** | | | |
| CP4 EPSPS 5' Probe F | AGCAGCATCCACGAGCTTAT | 59.98 | 709 bp |
| CP4 EPSPS 5' Probe R | ATGGGTTCAATCACGGTTGT | 60.08 |  |
| CP4 EPSPS 3' Probe F | GCGCTAATCTAACGGTCGAA | 60.33 | 737 bp |
| CP4 EPSPS 3' Probe R | TCAAGCGGCCTTAGTATCAGA | 59.96 |  |
| OsGRP3 3'UTR Probe F | CATCGTGGCCAGTTATCCTT | 59.93 | 554 bp |
| OsGRP3 3'UTR Probe R | TGCAAAATGGAAATGCTGTG | 60.63 |  |
