# Supplementary material for: Endogenous tassel-specific small RNAs-mediated RNA interference enables a novel glyphosate-inducible male sterility system for commercial production of hybrid seed in Zea mays L
Source: PLoS One. 2018 Aug 23;13(8):e0202921. doi: 10.1371/journal.pone.0202921 (PMC6107248; doi:10.1371/journal.pone.0202921)
Supplement: S2 Table — (DOCX) [file pone.0202921.s005.docx]

**S2 Table. Representative mts-siRNAs with sequence homology to the mts-siRNA target sequence.**

| **siRNA ID** | **Sequence** | **Complementary sequence** |
| --- | --- | --- |
| A | AUCACCAUGCACGGUCGU | ACGACCGUGCAUGGUGAU |
| B | GGAUUCAUCACCAUGCACGG | CCGUGCAUGGUGAUGAAUCC |
| C | GACGGCGAUGUGACAUGCGUG | CACGCAUGUCACAUCGCCGUC |
| D | ACGACGACCGUGCAUGGUGAUG | CAUCACCAUGCACGGUCGUCGU |
| E | ACGACCGUGCAUGGUGAU | AUCACCAUGCACGGUCGU |
| F | ACCAUAGAACUGCAAAUGUCCG | CGGACAUUUGCAGUUCUAUGGU |
| G | ACACCAUAGAACUGCAAAUGUC | GACAUUUGCAGUUCUAUGGUGU |
| H | ACGACGACCGUGCAUGGUG | CACCAUGCACGGUCGTCGU |
| I | GAGGACGGCGAUGUGACAUGCG | CGCAUGUCACAUCGCCGUCCUC |
| J | GACGGCGAUGUGACAUGCGUGU | ACACGCAUGUCACAUCGCCGUC |
