# Supplementary material for: Endogenous tassel-specific small RNAs-mediated RNA interference enables a novel glyphosate-inducible male sterility system for commercial production of hybrid seed in Zea mays L
Source: PLoS One. 2018 Aug 23;13(8):e0202921. doi: 10.1371/journal.pone.0202921 (PMC6107248; doi:10.1371/journal.pone.0202921)
Supplement: S3 Table — (DOCX) [file pone.0202921.s006.docx]

**S3 Table. Pollen yield per tassel and pollen viability.**

| **Treatment** | **No spray** | **V3 spray** | **V3, V8 and V10 spray** |
| --- | --- | --- | --- |
| **Plants** | Viable pollen (%) | Viable pollen (%) | Viable pollen (%) |
| **WT** | 98.77±0.03 | ND | ND |
| **Containing NK603** | 98.73±0.02 | 98.67±0.02 | 98.64±0.01 |
| **Containing MON 87429** | 98.69±0.02 | 98.15±0.05 | 0.00±0.00 |

Glyphosate sprays at 1.5, 0.75, and 0.75 lbs ae/acre were conducted at V3, V8, and V10, respectively. Ten anthers were collected from at least 8 plants per treatment of each entry for pollen staining. ND: no data because one glyphosate application effectively killed WT plants. Vn: leaf stages by number of leaves on the maize plant.
